# Biological Control and Insect Pathology

**DOI:** 10.3390/insects12040291

**Published:** 2021-03-27

**Authors:** Eustachio Tarasco, Francesca De Luca

**Affiliations:** 1 Department of Soil, Plant and Food Sciences, University of Bari “Aldo Moro”, Via Amendola 165/A, 70126 Bari, Italy; eustachio.tarasco@uniba.it; 2 Institute for Sustainable Plant Protection of CNR, Via Amendola 122/D, 70126 Bari, Italy

## 1. Introduction

Agro-forestry intensification is one of the main drivers of the global biodiversity crisis and decline in arthropods and particularly insects [1,2]. In addition, an impressive amount of research has been published regarding the detrimental effects of pesticides, which was the main approach developed for pest control in the previous century, but which has been progressively replaced by the use of Biological Control Agents (BCAs) and biopesticides to provide satisfactory pest management [3,4,5]. Microbial control is a growing sector within the framework of biological control and Integrated Pest Management (IPM). Methods need to be developed to avoid the widespread use of insecticides and to minimize effects on nontarget organisms. Studies collected in this Special Issue cover the use of biological control agents and entomopathogens, focusing on the fields of insect pathology and biological control and providing an update to the current EU Biological Control Panel (EPPO).

## 2. The Role of Biological Control Agents (BCAs) and Biopesticides

There are no sufficient BCAs and biopesticides to replace products that have been forbidden to use. Therefore, there is an even more urgent need to carry out research for the discovery and development of new BCAs and biopesticides. Current legislative efforts tend to support the registration of lower-risk pest management products such as BCAs and biopesticides [6]. The general public has a greater awareness for environmentally friendly products in agriculture and forestry [7]. Therefore, there is a greater expectation for organic and pesticide-free products. Agro-forestry practices, such as the use of pesticides that pollute soil, water and food, have forced the industry to evaluate current pest management practices and how pesticides affect environmental quality. Changes in consumer acceptance of BCAs and biopesticide technology are thus driving the need for such products, leading initiatives in the development of “green” technologies for food production.

## 3. Biological Control Agent (BCA) Exploration and Discovery

Natural biodiversity provides excellent opportunities for finding eco-friendly biocontrol agents, and in particular microbial strains with suitable traits for pest management [8]. Numerous discoveries of BCAs and microbial strains have taken place in research labs worldwide and many institutions may be in possession of potential biopesticide candidates. A systematic approach is needed for selection of appropriate potential biopesticides, including entomopathogenic fungi, bacteria, viruses and nematodes, evaluating above all how BCAs and biopesticides fit into IPM programs that currently encourage employing more than a single pest management intervention. The articles of this Special Issue are small pieces that will contribute to make BCAs and biopesticides a reality in future pest management strategies.

## Data Availability

Not applicable.
